# The Impact of Preinjury Use of Antiplatelet Drugs on Outcomes of Traumatic Brain Injury: A Systematic Review and Meta-Analysis

**DOI:** 10.3389/fneur.2022.724641

**Published:** 2022-02-07

**Authors:** Li Cheng, Gaoliang Cui, Rong Yang

**Affiliations:** Department of Medicine Rehabilitation, The First People's Hospital of Shangqiu City, Shangqiu, China

**Keywords:** antiplatelets, antithrombotic, blood thinners, head injury, mortality, intracranial hemorrhage, complications

## Abstract

**Objective:**

The study aimed to compare outcomes of traumatic brain injury (TBI) in patients on pre-injury antiplatelet drugs vs. those, not on any antiplatelet or anticoagulant drugs.

**Methods:**

PubMed, Embase, and Google Scholar databases were searched up to 15th May 2021. All cohort studies comparing outcomes of TBI between antiplatelet users vs. non-users were included.

**Results:**

Twenty studies were included. On comparison of data of 2,447 patients on pre-injury antiplatelet drugs with 4,814 controls, our analysis revealed no statistically significant difference in early mortality between the two groups (OR: 1.30 95% CI: 0.85, 1.98 *I*^2^ = 80% *p* = 0.23). Meta-analysis of adjusted data also revealed no statistically significant difference in early mortality between antiplatelet users vs. controls (OR: 1.24 95% CI: 0.93, 1.65 *I*^2^ = 41% *p* = 0.14). Results were similar for subgroup analysis of aspirin users and clopidogrel users. Data on functional outcomes was scarce and only descriptive analysis could be carried out. For the need for surgical intervention, pooled analysis did not demonstrate any statistically significant difference between the two groups (OR: 1.11 95% CI: 0.83, 1.48 *I*^2^ = 55% *p* = 0.50). Length of hospital stay (LOS) was also not found to be significantly different between antiplatelet users vs. non-users (MD: −1.00 95% CI: −2.17, 0.17 *I*^2^ = 97% *p* = 0.09).

**Conclusion:**

Our results demonstrate that patients on pre-injury antiplatelet drugs do not have worse early mortality rates as compared to patients, not on any antiplatelet or anticoagulant drugs. The use of antiplatelets is not associated with an increased need for neurosurgical intervention and prolonged LOS.

## Introduction

Traumatic brain injury (TBI) is often described as an insult due to a bump, blow or jolt to the head which leads to impairment of brain function (1). The spectrum of TBI can range from mild alteration in consciousness to a severe comatose state or death with or without evidence of intracranial hemorrhage (ICH) (2). It is an important medical, economic and social problem that affects all countries globally with an annual incidence rate of 349 per 100,000 person-years (3). TBI is commonly seen after falls or motor-vehicle accidents and it is one of the leading causes of mortality and disability in Western countries (4).

On account of the increasingly aging global population, there has been a rise in elderly patients sustaining TBI (5). An important aspect in treating older adults is to consider the associated comorbidities and the effect of several drugs to manage them (6). Antiplatelet and anticoagulant drugs are commonly prescribed after several conditions like myocardial infarction, interventional cardiac procedures, atrial fibrillation, knee or hip joint replacement, deep vein thrombosis, or pulmonary embolism to reduce the risk of systemic thromboembolism (7, 8). The use of such drugs complicates the management of TBI patients due to an increased risk of bleeding which may significantly alter patient prognosis (9, 10).

Of the two classes of antithrombotics, anticoagulants are known to cause greater alteration of the coagulation profile as compared to antiplatelet drugs. Indeed, studies have found that the risk of bleeding is significantly higher with anticoagulants as compared to antiplatelets alone (11). In the case of patients sustaining TBI, research suggests that preinjury use of anticoagulants (12) is a significant risk factor for developing ICH after TBI whereas corresponding evidence for antiplatelet drugs is ambiguous (13, 14). Recent meta-analysis studies have assessed the impact of preinjury anticoagulation on outcomes of general trauma and TBI patients and have noted significantly increased mortality in anticoagulant users as compared to controls (1, 10). However, similar evidence for antiplatelet drugs is scarce. To the best of our knowledge, only one meta-analysis published in 2013 has evaluated the role of pre-injury antiplatelet drugs on outcomes of TBI (15). The study reported that pre-injury antiplatelet users have a non-significant but slightly increased risk of mortality after TBI. An important limitation of the review was that only five studies were available for the pooled analysis. Since then, several researchers have published data on the outcomes of TBI with antiplatelet use and there is a need for more updated and comprehensive evidence (16–18). Therefore, we hereby aimed to conduct this systematic review and meta-analysis to assess the impact of preinjury use of antiplatelet drugs on outcomes of TBI.

## Materials and Methods

### Research Question

The predefined research question for the review was: Do outcomes of TBI patients on pre-injury antiplatelet drugs differ from those not on any anticoagulant or antiplatelet therapy?. We primarily aimed to assess the impact of antiplatelet drugs on the early and long-term mortality of patients with TBI. Secondary objectives were to assess the impact of antiplatelet drugs on the need for surgical intervention, functional outcomes, and length of hospital stay (LOS).

### Literature Search

This systematic review was conducted following the PRISMA statement (Preferred Reporting Items for Systematic Reviews and Meta-analyses) (19). We searched for eligible studies electronically on the databases of PubMed, Embase, and Google Scholar. Two reviewers carried out the literature search independent of each other. Search limits were from the inception of the databases to 15th May 2021. The main terms used for the literature search in various combinations were: “traumatic brain injury”, “head injury”, “intracranial hemorrhage”, “antiplatelets”, “antithrombotic”, “aspirin”, “clopidogrel”, “ticagrelor”, “dipyridamole”, “prasugrel”, and “eptifibatide”. Search strategy in detail is presented in Supplementary Table 1. After deduplication of the search results, we reviewed the output of each database by assessing the titles and abstracts of every study. We identified articles relevant to the review and extracted their full texts. The two reviewers independently evaluated these studies for final inclusion in the review. We resolved any disagreements by discussion. In the end, we reviewed the reference list of included studies for any missed references.

### Eligibility Criteria

We structured the eligibility criteria on the PICOS (Population, Intervention, Comparison, Outcome, Study type) framework. The detailed criteria were 1. Cohort studies were conducted on patients sustaining TBI (*Population*). 2. Studies comparing data of patients with preinjury use of an antiplatelet drug (*Intervention*) with a control group not on any antiplatelet or anticoagulant drugs (*Comparison*). 3. Studies assessing any one of the following outcomes: mortality, functional outcome, need for surgical intervention, LOS (*Outcomes*).

Exclusion criteria for the review were are follows: (1) Studies on trauma patients not reporting separate data for TBI, (2) Studies including patients on both anticoagulant and antiplatelet drugs and not reporting separate data for antiplatelet drugs, (3) Single arm studies not comparing outcomes with control group, (4) Non-English language studies, case reports, and review articles, (5) Studies reporting duplicate data. In case of two or more studies were from the same healthcare setup, we included the article with the largest sample size.

### Data Extraction and Quality Assessment

Data from each study was sourced by two authors independently. We extracted details of the first author, publication year, study type, country and study location, study period, included population, study groups, sample size, age, Glasgow coma scale (GCS), number of patients undergoing reversal of antiplatelet drugs, type of antiplatelet used and study outcomes.

The methodological quality of included studies was assessed using the Newcastle-Ottawa scale (NOS) (20). This too was carried out in duplicate and independently by two study investigators. Studies were awarded points for selection of study population, comparability, and outcomes. The maximum score which can be awarded is nine.

### Statistical Analysis

The meta-analysis was conducted using “Review Manager” (RevMan, version 5.3; Nordic Cochrane Centre [Cochrane Collaboration], Copenhagen, Denmark; 2014). We used a random-effects model for all outcomes. Early mortality was defined as events occurring within 30 days of injury. We pooled crude mortality rates and the need for surgical intervention using odds ratios (OR) with 95% confidence intervals (CI). We also extracted multivariable-adjusted data on mortality were available and pooled them using the generic inverse variance function of the software. Mean and standard deviation (SD) data of the length of hospital stay was extracted and pooled to calculate the mean difference (MD) and 95% CI. Median, range and interquartile range data was converted into mean and standard deviation (SD) when required using the method of Wan et al. (21). A sensitivity analysis was also performed for a meta-analysis of the primary outcome. Individual studies were sequentially excluded from the meta-analysis in the software itself to check any undue influence of the study on the total effect size. A sub-group was performed for crude mortality rates based on the type of antiplatelet drug.

Heterogeneity was assessed using the *I*^2^ statistic. *I*^2^ values of 25–50% represented low, values of 50–75% medium, and more than 75% represented substantial heterogeneity. We used funnel plots to assess publication bias for the primary outcome. *P* ≤ 0.05 was considered statistically significant.

## Results

### Details of Search and Included Studies

**Figure 1:**
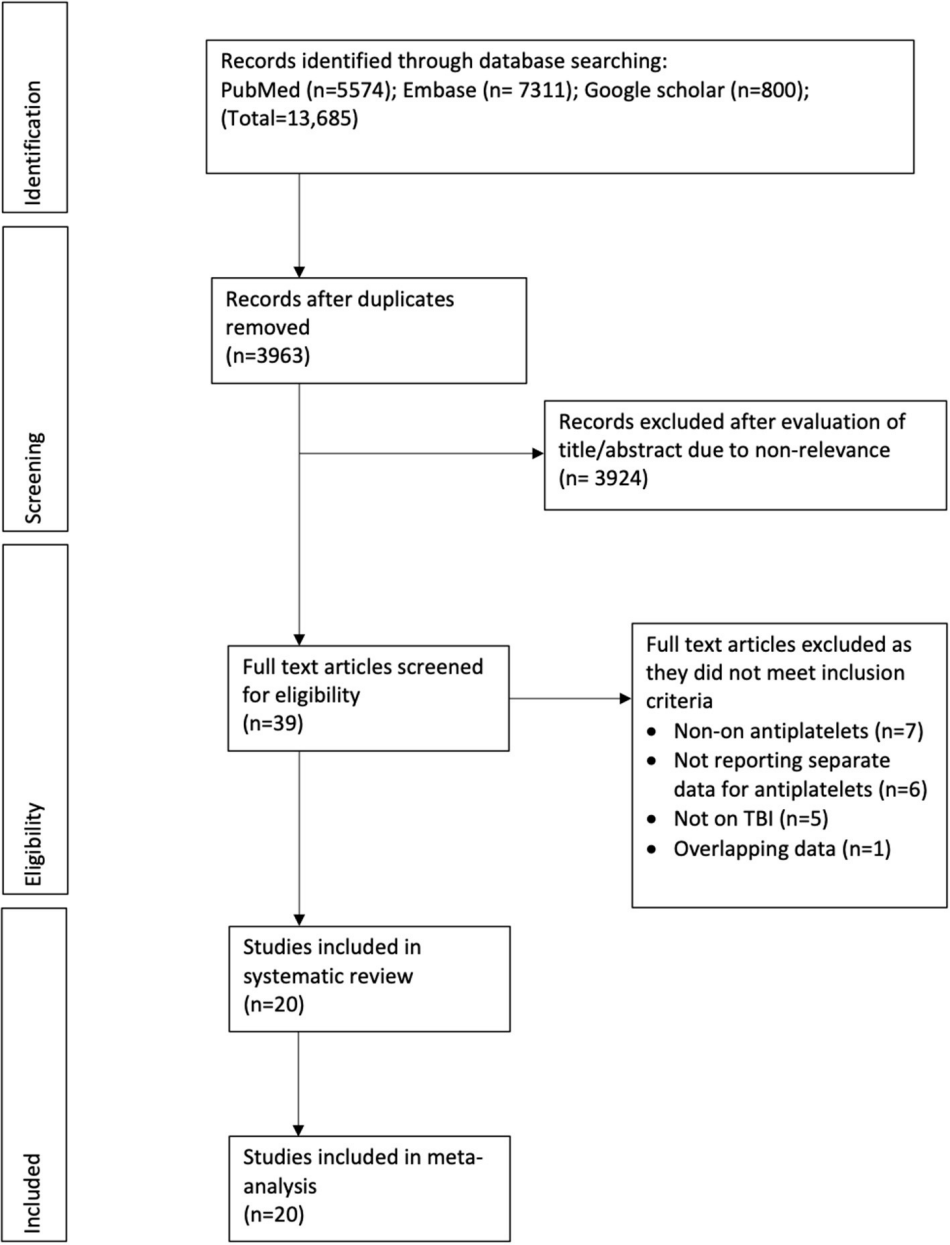
Study flow chart.

We identified a total of 3,678 unique articles after the literature search (Figure 1). Thirty-eight were selected for full-text analysis of which 19 were excluded as they did not fulfill the inclusion criteria. Finally, 20 studies were included in this review (16–18, 22–38). Characteristics of included studies are presented in Table 1. The studies were published between 2002 and 2021. The majority of them originated from the USA. Except for two (17, 27), all were retrospective cohort studies. While all studies included patients with varying degrees of TBI, two studies (31, 34) included only those patients who underwent neurosurgical intervention. The included patients were of the elderly age group in most studies. The sample size of the antiplatelet group varied from 19 to 833 patients while that of the control arm varied from 37 to 1,125 patients. Aspirin and clopidogrel were the most common antiplatelets used by the patients. Only four studies (18, 24, 29, 33) reported separate data for single and double antiplatelet users. In the remaining studies, a mix of single and double drug users was compared with controls. Eleven of the 19 studies had no data on reversal of antiplatelet drugs post-TBI. Assessing the quality of included studies, the NOS score varied from 6 to 8.

**Table 1:** Details of included studies.

**Study**	**Study type**	**Country**	**Study location**	**Study duration**	**Study population**	**Groups**	**Sample size**	**Age (years)**	**GCS**	**AP reversed**	**AP type**	**NOS score**
Ronning et al. (36)	RC	Norway	Oslo University Hospital Ullevål	2014–2019	≥65 years with TBI	AP No AP	267 360	NR	NR	NR	All types	8
Wettervik et al. (37)	RC	Sweden	Uppsala University	2008–2018	All patients with TBI	AP No AP	63 685	NR 49	NR 6 [5,6]	6	ASA and CLO	8
Robinson et al. (35)	RC	USA	University of Cincinnati and University of Pittsburgh Medical Center	2018	≥18 years with isolated head injury and non–comatose SDH	AP No AP	106 126	79.2 56.2	NR	37	ASA and CLO	8
Fernando et al. (38)	RC	Canada	The Ottawa Hospital network	2011–2016	≥18 years with mild TBI	AP No AP	50 201	NR	NR	NR	All types	8
Scotti et al. (18)	RC	Canada	Montreal general hospital	2014–2016	≥65 years with TBI	AP Dual AP No AP	413 48 641	NR	NR	144 16	ASA and CLO	8
Suehiro et al. (17)	PC	Japan	Multicentric	2015–2017	≥65 years with ICH	AP No AP	117 495	80.1 76.7	7.5 ± 4 6.8 ± 3.6	NR	NR	6
Tollefsen et al. (16)	RC	Norway	St.Olavs Hospital	2004–2013	≥50 years with TBI	AP No AP	43 121	77.3 63.7	9.5 [6–12] 11 [6–13]	3	ASA and CLO	8
Sumiyoshi et al. (33)	RC	Japan	National Disaster Medical Center	1995–2014	≥60 years with TBI	AP Dual AP No AP	283 81 570	73.5 74.3 73.1	12.2 ± 3.3 11.7 ± 3.6 11.9 ± 3.7	NR	ASA and CLO	8
Lee et al. (34)	RC	USA	Harborview Medical Center	2008–2012	≥65 years with ICH undergoing neurosurgery	AP No AP	87 84	78.3 75.9	12.8 ± 3.4 11.4 ± 4	38	ASA	8
Farsi et al. (32)	PC	Iran	Hazrat-e-Rasoul-e-Akram, Haft-e-Tir, and Firouzgar academic Hospitals	2013–2014	≥18 years with mild TBI	AP No AP	135 1005	NR	NR	NR	ASA and CLO	6
Okazaki et al. (30)	RC	Japan	Kagawa University Hospital	2008–2015	≥65 years with severe TBI	AP No AP	31 109	NR	NR	NR	NR	8
Han et al. (31)	RC	Korea	Dongguk University Ilsan Hospital	2006–2015	Undergoing decompressive craniectomy for TBI	AP No AP	19 71	62.4 55.1	8.8 ± 3.3 9.2 ± 3	NR	ASA and CLO	6
Grandhi et al. (29)	RC	USA	University of Pittsburgh Medical Center Presbyterian Hospital	2006–2010	≥65 years with TBI	ASA CLO Dual AP No AP	543 97 193 501	79.8 80 80 79.8	15 [14–15] 15 [14–15] 15 [14–15] 15 [13–15]	271 81 165	ASA and CLO	8
Cull et al. (28)	RC	USA	John H. Stroger Hospital and Carle Foundation Hospital	2008–2011	≥65 years with blunt head trauma	AP No AP	422 1125	66.1 57.3	NR	NR	ASA and CLO	8
Peck et al. (26)	RC	USA	Scripps Mercy Hospital	2006–2011	≥55 years with blunt force TBI	AP No AP	38 273	77.3 74.9	13.4 ± 3.3 13.4 ± 3.1	NR	CLO and DIP	8
Joseph et al. (27)	PC	USA	University of Arizona	2011–2012	All patients with TBI	AP No AP	71 71	71,6 69.8	14 (3–15) 14 (3–15)	71	CLO	8
Wong et al. (23)	RC	USA	Queens Medical Center	2001–2005	All patients with TBI	ASA CLO No AP	90 21 178	67.3 71.5 65.5	NR	19 0	ASA and CLO	8
Ivascu et al. (25)	RC	USA	William Beaumont Hospital	1999–2004	All patients with ICH	AP No AP	109 42	77 76	13.6 ± 2.8 13.1 ± 2.9	40	ASA and CLO	6
Fortuna et al. (24)	RC	USA	University of Cincinnati	2003–2005	≥55 years with hemorrhagic brain injury	ASA CLO Dual AP No AP	91 17 18 250	71.9 75.1 76.4 66.4	12.5 ± 0.4 11.8 ± 1.3 13.3 ± 0.8 12 ± 0.3	NR	ASA and CLO	6
Mina et al. (22)	RC	USA	William Beaumont Hospital	1997–1998	All patients with intracranial injuries	AP No AP	19 37	74 75	11.8 ± 4.3 12.2 ± 2.6	NR	ASA	6

*GCS, Glasgow coma scale; AP, antiplatelet; ASA, aspirin; CLO, clopidogrel; ICH, intracranial hemorrhage; SDH, subdural hemorrhage; TBI, traumatic brain injury; NR, no reported; NOS, Newcastle Ottawa Scale*.

### Mortality

**Figure 2:**
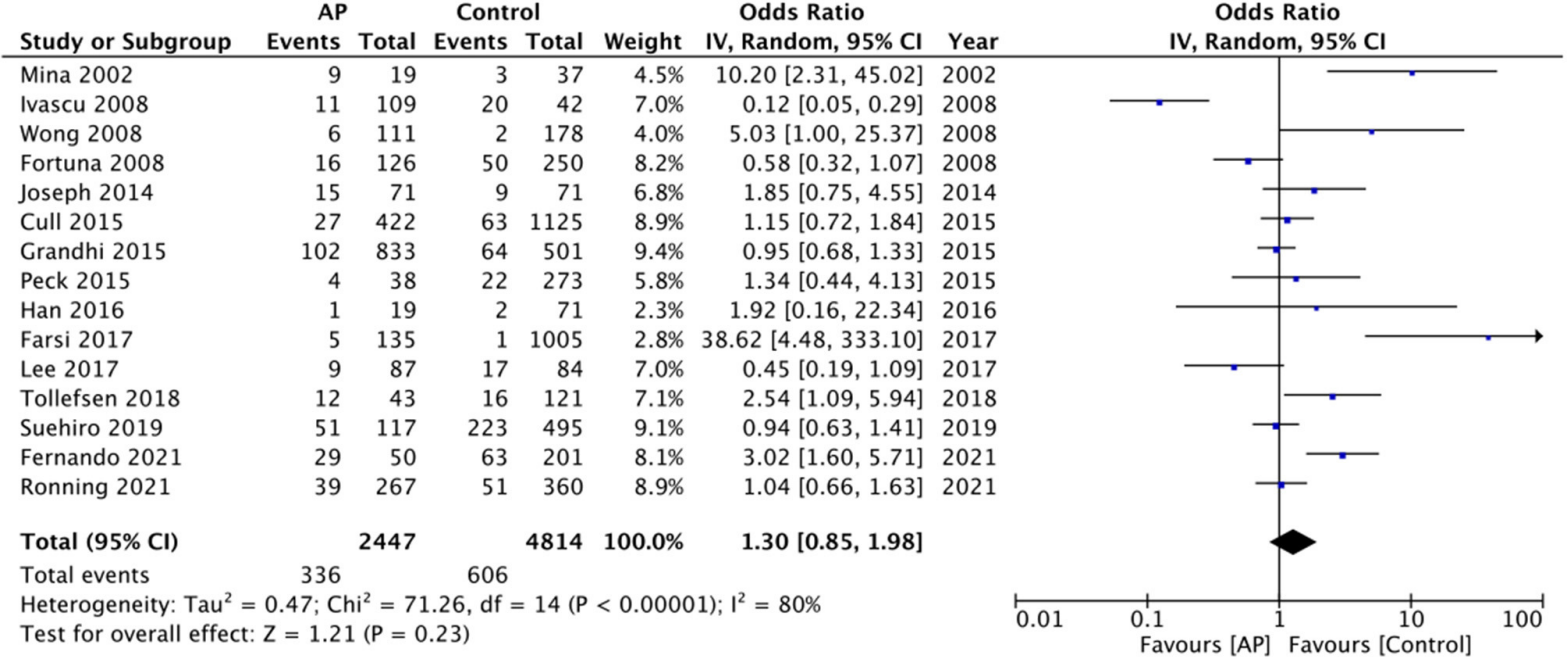
Meta-analysis of crude early mortality rates between antiplatelet users vs. controls.

**Figure 3:**
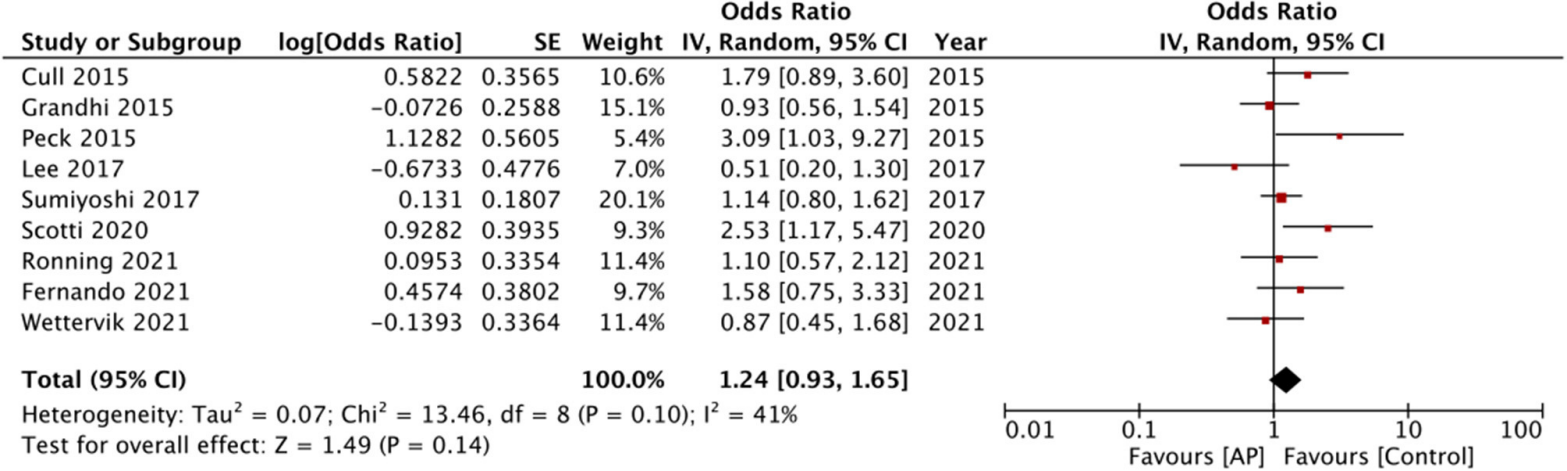
Meta-analysis of adjusted early mortality rates between antiplatelet users vs. controls.

Fifteen studies reported crude early mortality rates. On comparison of data of 2,447 patients on pre-injury antiplatelet drugs with 4,814 controls, our analysis revealed no statistically significant difference in early mortality between the two groups (OR: 1.30 95% CI: 0.85, 1.98 *I*^2^ = 80% *p* = 0.23) (Figure 2). There was no evidence of publication bias (Supplementary Figure 1). On sensitivity analysis, we found no change in the significance of the results on the exclusion of any study. Nine studies reported multivariable-adjusted data on early mortality. Meta-analysis revealed no statistically significant difference between antiplatelet users vs. controls (OR: 1.24 95% CI: 0.93, 1.65 *I*^2^ = 41% *p* = 0.14) (Figure 3). Results were stable on sensitivity analysis and did not change on the exclusion of any study.

**Figure 4:**
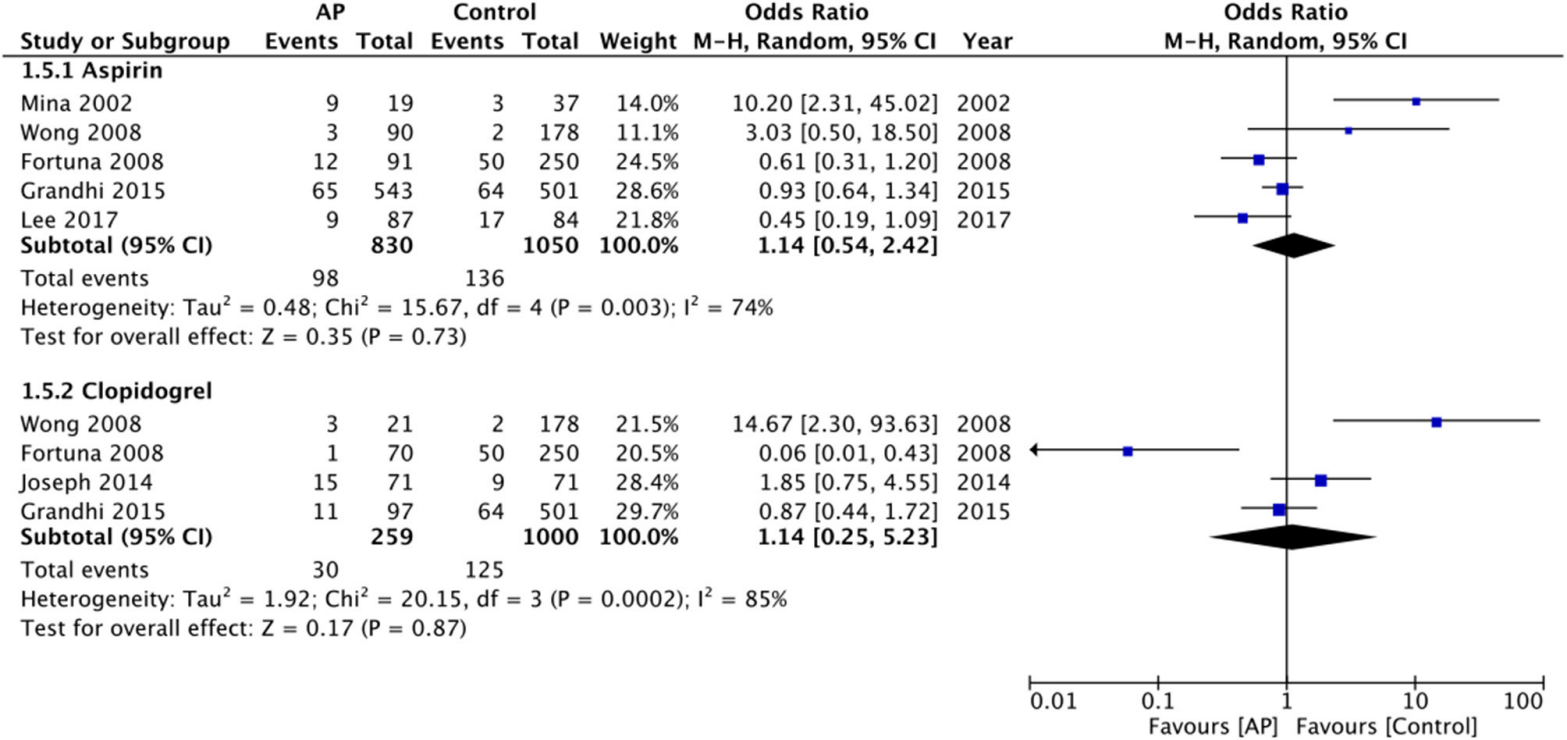
Meta-analysis of crude early mortality rates between antiplatelet users vs. controls based on the type of drug.

Separate data for aspirin and clopidogrel could be extracted from only five and four studies, respectively. Pooled analysis failed to demonstrate any difference in mortality rates for aspirin (OR: 1.14 95% CI: 0.54, 2.42 *I*^2^ = 74% *p* = 0.73) or for clopidogrel (OR: 1.14 95% CI: 0.25, 5.23 *I*^2^ = 85% *p* = 0.87) (Figure 4). Due to a lack of data, we could not analyze the impact of antiplatelet drugs on long-term mortality rates.

### Functional Outcomes

Due to limited data and variable reporting amongst included studies, pooled analysis for functional outcomes could not be carried out. Results were analyzed descriptively. Robinson et al. (35) in their analysis reported poor functional outcomes (defined as a score of ≥2 on the modified Rankin Scale) at discharge in 58% of antiplatelet users as compared to 33% controls. On multivariate analysis, antiplatelet use was not found to be a significant predictor of poor functional outcomes. Okazaki et al. (30) also reported that antiplatelet use was not independently associated with unfavorable outcomes at discharge (defined as Glasgow outcome scale [GOS] ≤ 4) (OR: 1.33 95% CI: 0.30–5.96). Similarly, Sumiyoshi et al. (33) have reported that antiplatelet use is not associated with unfavorable outcomes (GOS <4) (OR: 1.74 95% CI: 0.87–3.47). On the other hand, Scotti et al. (18) in their study found that both single antiplatelet use (OR: 1.58 95% CI: 1.01–2.49) and double antiplatelet use (OR: 3.37 95% CI: 1.52–7.45) were significant predictors of functional dependency at discharge (GOS ≤ 4). Farsi et al. (32) noted that antiplatelet users had a higher risk of moderate (5.2% vs. 1.4%) and severe disability (2.2% vs. 0.2%) as compared to controls.

### Need for Surgical Intervention and LOS

**Figure 5:**
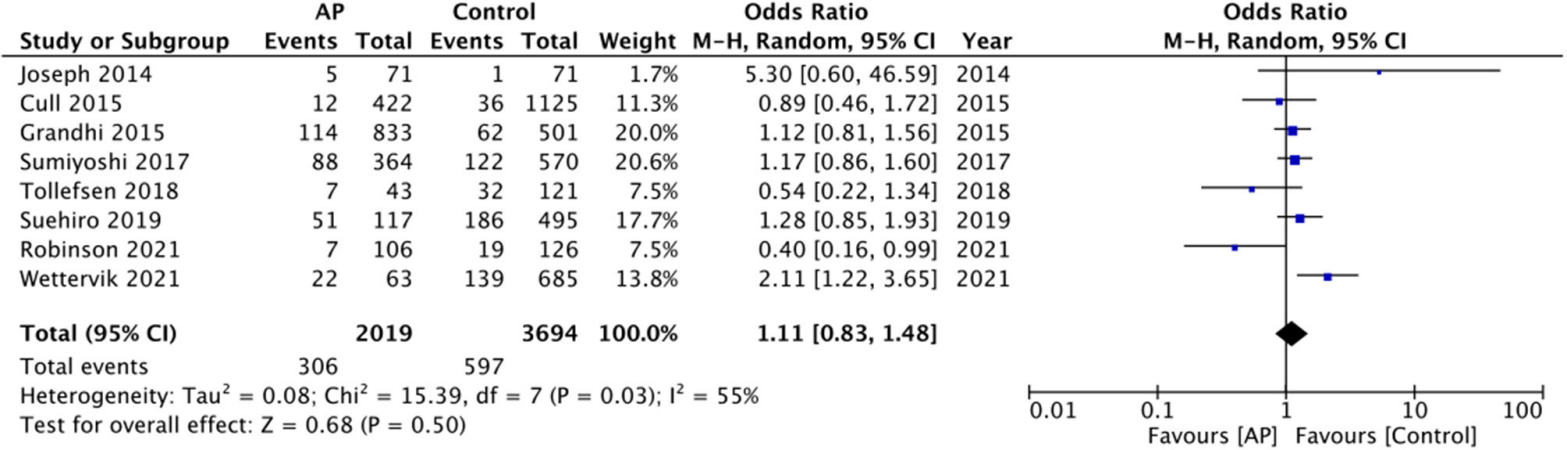
Meta-analysis of need for surgical intervention between antiplatelet users vs. controls.

**Figure 6:**
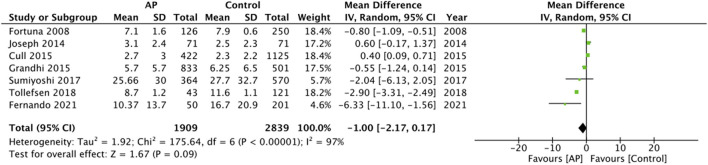
Meta-analysis of length of hospital stay between antiplatelet users vs. controls.

Data on the need for surgical intervention was available from eight studies. The pooled analysis did not demonstrate any statistically significant difference between the two groups (OR: 1.11 95% CI: 0.83, 1.48 *I*^2^ = 55% *p* = 0.50) (Figure 5). LOS was also not found to be significantly different between antiplatelet users vs. non-users (MD: −1.00 95% CI: −2.17, 0.17 *I*^2^ = 97% *p* = 0.09) (Figure 6).

## Discussion

Much research has been conducted on the impact of preinjury use of antiplatelet and anticoagulant drugs on trauma patients (9, 10). Indeed, with a flourishing use of these medications for multiple systemic indications, a large number of trauma patients are being treated under the influence of antithrombotics. In this context, it is essential to gauge the impact of these medications on patient outcomes so that appropriate and timely diagnostic and treatment measures can be undertaken to improve patient survival and functional outcomes. The current study aims to fill this gap by providing evidence on the impact of antiplatelet drugs on outcomes of TBI patients.

In the case of patients with a head injury, any TBI leading to ICH can be a devastating and life-threatening complication (39). ICH may develop immediately or late after the initial injury and can significantly alter the patient's prognosis (40). Theoretically, any patient on antithrombotics would have an increased risk of ICH post-TBI. However, while the risk of intracranial complications post TBI is well established for oral anticoagulants (12), the role of antiplatelet drugs has been controversial. Savioli et al. (14) in a recent study involving 483 patients on antiplatelet drugs sustaining minor head injury noted no increased risk of ICH amongst antiplatelet users. Contrarily, a 2017 meta-analysis by Brand et al. (13) noted a 1.87 times increased risk of ICH in antiplatelet users sustaining a head injury. It is important to note that the severity of ICH is an important factor affecting prognosis (41). Research has indicated that preinjury use of anticoagulants is associated with increased intracranial hematoma volume and hematoma expansion as compared to patients, not under any anticoagulant therapy and both these factors can influence overall mortality rates (42). However, the same has not been proven in the case of antiplatelet drugs. An animal study has shown that antiplatelet drugs do not increase hematoma volume and have no impact on functional outcomes after experimental ICH (43). Murthy et al. (44) in a recent study on primary intracerebral hemorrhage patients have demonstrated that prior use of antiplatelets does not affect hematoma volume, hematoma expansion, the incidence of major disability, or death.

In line with these studies, our primary analysis demonstrated that preinjury use of antiplatelet drugs has no impact on early mortality rates in the case of patients with TBI. The analysis is strengthened by the fact that no study had an undue effect on the overall results of sensitivity analysis. We also noted no difference in the need for neurosurgical intervention and LOS between antiplatelet users vs. controls. Our study was unable to conduct a quantitative analysis for functional outcomes, but limited data indicate that antiplatelet drugs may not have an adverse impact on functional outcomes as well. The results of our analysis differ from the past review on this subject which noted a slightly increased risk of mortality in antiplatelet users (15). By adding 15 more studies, our review is a significant update that clarifies the impact of these drugs on outcomes of TBI patients. In comparison with other antithrombotics, Lim et al. (1) in a meta-analysis have noted an increased risk of overall mortality amongst TBI patients on preinjury anticoagulant drugs. Similar to our results, preinjury anticoagulants were not associated with an increased need for neurosurgical intervention or prolonged LOS in patients with TBI. In the case of general trauma, Lee et al. (10) in a pooled analysis of 1,365,446 patients have also demonstrated that pre-injury anticoagulation significantly increases the risk of overall mortality but does not impact the incidence of surgical intervention. This disparity between antiplatelets and antithrombotics on patient outcomes also has been noted by a recent study by Narula et al. (45) wherein only preinjury anticoagulants were found to increase mortality of trauma patients but not preinjury antiplatelets.

The differential impact of antiplatelets and anticoagulants on overall mortality in TBI patients corroborates with the contrasting evidence on the effect of these drugs on ICH severity. It is plausible that since anticoagulants are known to increase the risk of ICH and its severity in TBI unlike antiplatelets, they have a greater impact on patient prognosis as compared to antiplatelets (42, 44). It is also important to note that the studies included in this review had a heterogeneous population and patients with varying degrees of TBI were included. Nevertheless, there were no major variations in Glasgow Coma Scale (GCS) scores between antiplatelet and non-antiplatelet groups. As randomized trials are not possible to assess the impact of antiplatelets on trauma patients, one must interpret the results because there were baseline differences between the study and control groups. However, one of the strengths of our review is that the results on mortality were reiterated on a pooled analysis of adjusted data, albeit from only eight studies.

Aspirin and clopidogrel were the most common antiplatelet drugs used in the included studies. Both the drugs inhibit platelet aggregation but by different mechanisms. While aspirin acts by inhibiting cyclooxygenase enzymes and reducing the production of thromboxane A2, clopidogrel acts by inhibiting adenosine dinucleotide phosphate (ADP) receptors and subsequent ADP-mediated activation of the glycoprotein GPIIb/IIIa complex (46). Clopidogrel is considered to be superior to aspirin for the prevention of thromboembolic events and is being more widely used (47). However, we noted no difference between the two drugs for their impact on mortality in TBI patients.

Another important aspect to consider is the difference between dual antiplatelet vs. single antiplatelet therapy. In the majority of the included studies, data of single and dual antiplatelet therapy were combined and compared with controls. Due to scarce data, we could not differentiate the mortality rates with these two regimens. Research indicates that as compared to single antiplatelet drugs, dual antiplatelet therapy reduces the risk of thromboembolic events in ischemic stroke patients but with a significant increase in the risk of bleeding (48). In one of the included studies, Scotti et al. (18) have demonstrated that dual antiplatelet but not single antiplatelet therapy is associated with a significantly increased risk of mortality in TBI patients. Similarly, Sumiyoshi et al. (33) also noted an increased risk of mortality with dual as compared to single antiplatelet therapy. Further studies reporting separate data are needed to clarify the impact of dual antiplatelet therapy on outcomes of TBI.

As past evidence indicated higher mortality in antiplatelet users sustaining TBI (15), the use of platelet transfusion has been practiced to improve outcomes of patients with TBI (49). In the included studies, data on antiplatelet reversal was not universally reported. Furthermore, there was significant variability in the number of patients transfused with platelets where data was available. However, recent reviews have suggested that there is no clear evidence on the benefits of platelet transfusions in TBI patients on prior antiplatelet drugs (50, 51). On the contrary, reversal of antiplatelet agents may lead to a non-significant increase in thromboembolic events (51).

Our review has some limitations. Foremost, all included studies were retrospective in nature and may be prone to selection bias. Baseline matching was not carried out by majority studies. Furthermore, multivariable-adjusted outcomes were also not reported by several studies. Since antiplatelet drugs are usually prescribed in patients with comorbidities it is plausible that several confounding factors could have influenced outcomes. Secondly, due to a lack of data were unable to assess the impact of antiplatelet drugs on functional outcomes. It is also unclear if there is any difference in long-term outcomes with these drugs. Thirdly, an important limitation of our meta-analysis is that data of single and dual antiplatelet therapy were combined and compared with controls. Since a few studies have demonstrated worse outcomes with dual antiplatelet therapy, future studies need to report separate outcomes of these regimens. Fourthly, the majority of data in our review was on aspirin and clopidogrel. It is not known how other antiplatelets like ticagrelor, dipyridamole, prasugrel impact outcomes of TBI. Lastly, the studies in our meta-analysis included a mix of TBI patients (mild, moderate and severe). Specifically, one study included only severe TBI patients (17) while another study included only mild TBI patients (29). A subgroup analysis or a meta-regression analysis assessing the relationship between TBI severity and mortality rates could not be conducted as close to 50% of the studies did not report baseline GCS scores. Furthermore, the mean and SD of GCS scores of the remaining studies were wide and they could not be divided into specific subgroups for a separate analysis. We could assess the influence of these studies only via a sensitivity analysis which did not change the study results. However, there is a need for future studies to take this variable into account while reporting outcomes.

## Conclusions

Our results demonstrate that patients on pre-injury antiplatelet drugs do not have worse early mortality rates as compared to patients, not on any antiplatelet or anticoagulant drugs. The use of antiplatelets is not associated with an increased need for neurosurgical intervention and prolonged LOS. Further studies with baseline matching or reporting adjusted data are needed to strengthen current conclusions.

## Data Availability Statement

The raw data supporting the conclusions of this article will be made available by the authors, without undue reservation.

## Author Contributions

LC and GC conceived and designed the study and were involved in literature search and data collection. LC and RY analyzed the data. LC and GC wrote the paper. GC and RY reviewed and edited the manuscript. All authors have read and approved the final manuscript.

## Conflict of Interest

The authors declare that the research was conducted in the absence of any commercial or financial relationships that could be construed as a potential conflict of interest.

## Publisher's Note

All claims expressed in this article are solely those of the authors and do not necessarily represent those of their affiliated organizations, or those of the publisher, the editors and the reviewers. Any product that may be evaluated in this article, or claim that may be made by its manufacturer, is not guaranteed or endorsed by the publisher.
