# Supplementary material for: The Impact of Preinjury Use of Antiplatelet Drugs on Outcomes of Traumatic Brain Injury: A Systematic Review and Meta-Analysis
Source: Front Neurol. 2022 Feb 7;13:724641. doi: 10.3389/fneur.2022.724641 (PMC8858945; doi:10.3389/fneur.2022.724641)
Supplement: Supplementary file 1 [file Table_1.DOCX]

Supplementary Table 1: Search strategy

| **Search number** | **Query** | **Search Details** |
| --- | --- | --- |
| **1** | (traumatic brain injury) AND (antiplatelets) | ("brain injuries, traumatic"[MeSH Terms] OR ("brain"[All Fields] AND "injuries"[All Fields] AND "traumatic"[All Fields]) OR "traumatic brain injuries"[All Fields] OR ("traumatic"[All Fields] AND "brain"[All Fields] AND "injury"[All Fields]) OR "traumatic brain injury"[All Fields]) AND ("antiplatelet"[All Fields] OR "antiplatelets"[All Fields]) |
| **2** | (head injury) AND (antiplatelets) | ("craniocerebral trauma"[MeSH Terms] OR ("craniocerebral"[All Fields] AND "trauma"[All Fields]) OR "craniocerebral trauma"[All Fields] OR ("head"[All Fields] AND "injury"[All Fields]) OR "head injury"[All Fields]) AND ("antiplatelet"[All Fields] OR "antiplatelets"[All Fields]) |
| **3** | (intracranial haemorrhage) AND (antiplatelet) | ("intracranial haemorrhage"[All Fields] OR "intracranial hemorrhages"[MeSH Terms] OR ("intracranial"[All Fields] AND "hemorrhages"[All Fields]) OR "intracranial hemorrhages"[All Fields] OR ("intracranial"[All Fields] AND "hemorrhage"[All Fields]) OR "intracranial hemorrhage"[All Fields]) AND ("antiplatelet"[All Fields] OR "antiplatelets"[All Fields]) |
| **4** | (traumatic brain injury) AND (antithrombotics) | ("brain injuries, traumatic"[MeSH Terms] OR ("brain"[All Fields] AND "injuries"[All Fields] AND "traumatic"[All Fields]) OR "traumatic brain injuries"[All Fields] OR ("traumatic"[All Fields] AND "brain"[All Fields] AND "injury"[All Fields]) OR "traumatic brain injury"[All Fields]) AND ("antithrombotic"[All Fields] OR "antithrombotics"[All Fields]) |
| **5** | (head injury) AND (antithrombotics) | ("craniocerebral trauma"[MeSH Terms] OR ("craniocerebral"[All Fields] AND "trauma"[All Fields]) OR "craniocerebral trauma"[All Fields] OR ("head"[All Fields] AND "injury"[All Fields]) OR "head injury"[All Fields]) AND ("antithrombotic"[All Fields] OR "antithrombotics"[All Fields]) |
| **6** | (intracranial haemorrhage) AND (antithrombotics) | ("intracranial haemorrhage"[All Fields] OR "intracranial hemorrhages"[MeSH Terms] OR ("intracranial"[All Fields] AND "hemorrhages"[All Fields]) OR "intracranial hemorrhages"[All Fields] OR ("intracranial"[All Fields] AND "hemorrhage"[All Fields]) OR "intracranial hemorrhage"[All Fields]) AND ("antithrombotic"[All Fields] OR "antithrombotics"[All Fields]) |
| **7** | ((aspirin) OR (clopidogrel)) AND (traumatic brain injury) | ("aspirin"[MeSH Terms] OR "aspirin"[All Fields] OR "aspirins"[All Fields] OR "aspirin s"[All Fields] OR "aspirine"[All Fields] OR ("clopidogrel"[MeSH Terms] OR "clopidogrel"[All Fields] OR "clopidogrel s"[All Fields])) AND ("brain injuries, traumatic"[MeSH Terms] OR ("brain"[All Fields] AND "injuries"[All Fields] AND "traumatic"[All Fields]) OR "traumatic brain injuries"[All Fields] OR ("traumatic"[All Fields] AND "brain"[All Fields] AND "injury"[All Fields]) OR "traumatic brain injury"[All Fields]) |
| **8** | ((aspirin) OR (clopidogrel)) AND (head injury) | ("aspirin"[MeSH Terms] OR "aspirin"[All Fields] OR "aspirins"[All Fields] OR "aspirin s"[All Fields] OR "aspirine"[All Fields] OR ("clopidogrel"[MeSH Terms] OR "clopidogrel"[All Fields] OR "clopidogrel s"[All Fields])) AND ("craniocerebral trauma"[MeSH Terms] OR ("craniocerebral"[All Fields] AND "trauma"[All Fields]) OR "craniocerebral trauma"[All Fields] OR ("head"[All Fields] AND "injury"[All Fields]) OR "head injury"[All Fields]) |
| **9** | ((aspirin) OR (clopidogrel)) AND (intracranial haemorrhage) | ("aspirin"[MeSH Terms] OR "aspirin"[All Fields] OR "aspirins"[All Fields] OR "aspirin s"[All Fields] OR "aspirine"[All Fields] OR ("clopidogrel"[MeSH Terms] OR "clopidogrel"[All Fields] OR "clopidogrel s"[All Fields])) AND ("intracranial haemorrhage"[All Fields] OR "intracranial hemorrhages"[MeSH Terms] OR ("intracranial"[All Fields] AND "hemorrhages"[All Fields]) OR "intracranial hemorrhages"[All Fields] OR ("intracranial"[All Fields] AND "hemorrhage"[All Fields]) OR "intracranial hemorrhage"[All Fields]) |
| 10 | ((((ticagrelor) OR (dipyridamole)) OR (prasugrel)) OR (eptifibatide)) AND (traumatic brain injury) | ("ticagrelor"[MeSH Terms] OR "ticagrelor"[All Fields] OR ("dipyridamol"[All Fields] OR "dipyridamole"[MeSH Terms] OR "dipyridamole"[All Fields]) OR ("prasugrel hydrochloride"[MeSH Terms] OR ("prasugrel"[All Fields] AND "hydrochloride"[All Fields]) OR "prasugrel hydrochloride"[All Fields] OR "prasugrel"[All Fields] OR "prasugrel s"[All Fields]) OR ("eptifibatide"[MeSH Terms] OR "eptifibatide"[All Fields])) AND ("brain injuries, traumatic"[MeSH Terms] OR ("brain"[All Fields] AND "injuries"[All Fields] AND "traumatic"[All Fields]) OR "traumatic brain injuries"[All Fields] OR ("traumatic"[All Fields] AND "brain"[All Fields] AND "injury"[All Fields]) OR "traumatic brain injury"[All Fields]) |
| 11 | ((((ticagrelor) OR (dipyridamole)) OR (prasugrel)) OR (eptifibatide)) AND (head injury) | ("ticagrelor"[MeSH Terms] OR "ticagrelor"[All Fields] OR ("dipyridamol"[All Fields] OR "dipyridamole"[MeSH Terms] OR "dipyridamole"[All Fields]) OR ("prasugrel hydrochloride"[MeSH Terms] OR ("prasugrel"[All Fields] AND "hydrochloride"[All Fields]) OR "prasugrel hydrochloride"[All Fields] OR "prasugrel"[All Fields] OR "prasugrel s"[All Fields]) OR ("eptifibatide"[MeSH Terms] OR "eptifibatide"[All Fields])) AND ("craniocerebral trauma"[MeSH Terms] OR ("craniocerebral"[All Fields] AND "trauma"[All Fields]) OR "craniocerebral trauma"[All Fields] OR ("head"[All Fields] AND "injury"[All Fields]) OR "head injury"[All Fields]) |
| 12 | ((((ticagrelor) OR (dipyridamole)) OR (prasugrel)) OR (eptifibatide)) AND (intracranial haemorrhage) | ("ticagrelor"[MeSH Terms] OR "ticagrelor"[All Fields] OR ("dipyridamol"[All Fields] OR "dipyridamole"[MeSH Terms] OR "dipyridamole"[All Fields]) OR ("prasugrel hydrochloride"[MeSH Terms] OR ("prasugrel"[All Fields] AND "hydrochloride"[All Fields]) OR "prasugrel hydrochloride"[All Fields] OR "prasugrel"[All Fields] OR "prasugrel s"[All Fields]) OR ("eptifibatide"[MeSH Terms] OR "eptifibatide"[All Fields])) AND ("intracranial haemorrhage"[All Fields] OR "intracranial hemorrhages"[MeSH Terms] OR ("intracranial"[All Fields] AND "hemorrhages"[All Fields]) OR "intracranial hemorrhages"[All Fields] OR ("intracranial"[All Fields] AND "hemorrhage"[All Fields]) OR "intracranial hemorrhage"[All Fields]) |
