# Supplementary figures and images for: The Impact of Preinjury Use of Antiplatelet Drugs on Outcomes of Traumatic Brain Injury: A Systematic Review and Meta-Analysis
Source: Front Neurol. 2022 Feb 7;13:724641. doi: 10.3389/fneur.2022.724641 (PMC8858945; doi:10.3389/fneur.2022.724641)

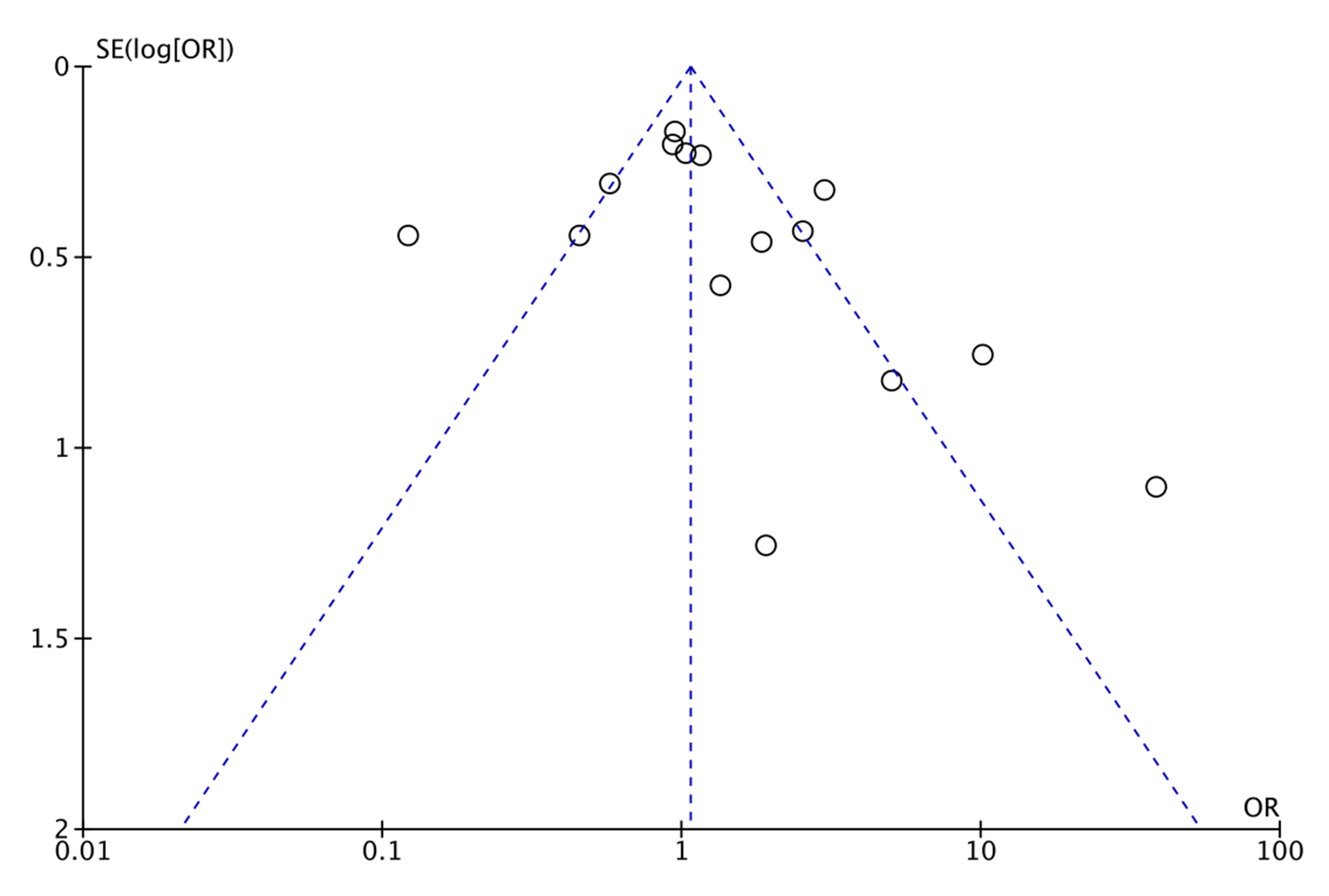

Supplement: Supplementary file 2 [file Image_1.TIFF]
